# From Diet to Disease: The Role of the Gut Microbiome and Microbial Metabolites in Horses

**DOI:** 10.3390/vetsci13080823

**Published:** 2026-08-18

**Authors:** Yuhong Wu, Yuhui Ma, Yiyi Zan, Xu Bai, Jing Li, Hai Li

**Affiliations:** 1 Department of Clinical Veterinary Medicine, College of Veterinary Medicine, China Agricultural University, Beijing 100193, China; wyh999@cau.edu.cn (Y.W.); zan11@cau.edu.cn (Y.Z.); 2 National Postdoctoral Research Workstation, Zhaosu Xiyu Horse Industry Co., Ltd., Yili 835699, China; mayuhuim@126.com; 3 Beijing Zhongnongda Veterinary Hospital Co., Ltd., Beijing 100193, China; 4 China Horse Industry Association, Beijing 100125, China; baixu@chinahorse.org; 5 State Key Laboratory of Veterinary Public Health Safety, Beijing 100193, China; 6 Dongfangmadu Equine Teaching Hospital, College of Veterinary Medicine, China Agricultural University, Beijing 100193, China

**Keywords:** equine microbiome, microbial metabolites, gut-lung axis, precision nutrition

## Abstract

The equine gastrointestinal microbiome plays an essential role in digestion, energy metabolism, immune regulation, and maintenance of intestinal homeostasis. Increasing evidence suggests that microbial-derived metabolites provide a functional link between diet, the microbiome, and host physiology. This narrative review summarizes current knowledge on how dietary factors, including structural carbohydrates, non-structural carbohydrates, protein, and lipids, influence microbial function and metabolite production in horses. Relevant publications available up to June 2026 were identified through searches of PubMed, Web of Science, Scopus, and Google Scholar and were narratively synthesized according to dietary factors, microbial metabolites, associated diseases, and nutritional interventions. Emphasis is placed on major microbial metabolites, including short-chain fatty acids, endotoxins, bile acid derivatives, tryptophan metabolites, and nitrogenous fermentation products, and their potential roles in health and disease. Current evidence supports a central role for the nutrition–microbiota–metabolite axis in gastrointestinal disorders such as colic, colitis, equine gastric ulcer syndrome, and carbohydrate-associated laminitis, whereas its involvement in equine metabolic syndrome and the gut–lung axis remains less well defined. The review also discusses microbiome-targeted nutritional interventions and emerging multi-omics approaches that are improving our understanding of microbial function. Collectively, these findings highlight the potential of microbiome-informed nutritional strategies to support equine health, welfare, disease prevention, and athletic performance.

## Simple Summary

The intestinal microbial community of horses plays a critical role in digestion, energy production, immune function, and overall health. Although early equine microbiome studies primarily described microbial community composition, growing evidence suggests that microbial metabolites are the important functional mediators of host–microbiome interactions and may better explain the biological consequences of dietary and environmental influences. This review summarizes current knowledge on how diet shapes microbial activity and the production of biologically active compounds in the equine gastrointestinal tract. Attention is given to microbial products that influence intestinal health, inflammation, metabolism, and respiratory function. Current evidence indicates that disturbances in the interactions among diet, gut microorganisms, and their metabolic products are associated with gastrointestinal disorders, metabolic syndrome, laminitis, and potentially respiratory disease. The review also discusses emerging nutritional strategies aimed at supporting beneficial microbial function and highlights future opportunities for individualized nutrition based on microbiome and metabolite profiling. A better understanding of these interactions may contribute to improved disease prevention, animal welfare, and athletic performance in horses.

## 1. Introduction

The horse is a hindgut-fermenting herbivore whose digestive physiology is uniquely adapted to the microbial fermentation of structural carbohydrates within the cecum and large colon [1,2,3,4]. Unlike ruminants, horses rely on an extensive hindgut microbial ecosystem to degrade plant polysaccharides that escape enzymatic digestion in the foregut [1,2,3,5]. This microbial community, composed primarily of bacteria, fungi, archaea, and viruses, functions as a metabolically active organ that contributes substantially to nutrient utilization, energy metabolism, and gastrointestinal homeostasis [1,4,6]. Fermentation of dietary substrates by the hindgut microbiota produces a diverse array of bioactive compounds that influence both local intestinal function and systemic physiology [4,5,6,7].

Advances in next-generation sequencing and microbiome research have substantially expanded our understanding of the equine gastrointestinal ecosystem [8]. Early investigations primarily focused on describing microbial composition and identifying taxonomic changes associated with diet, management practices, exercise, and disease [1]. Although these studies have provided important insights into the structure of the equine microbiome, microbial composition alone offers limited information regarding biological function [3,4,9]. Increasing evidence suggests that microbial metabolites provide a more direct functional readout of host–microbiome interactions than microbial abundance alone [8]. Consequently, equine microbiome research has progressively shifted from a composition-centered paradigm toward a function-centered framework that emphasizes microbial activity, metabolic outputs, and host responses [3,4,8,9].

Among the numerous factors influencing microbial function, diet represents the most important and readily modifiable determinant of the equine microbiome [2,4,10,11]. Structural carbohydrates derived from forage-based diets promote fibrolytic fermentation and support hindgut stability, whereas excessive intake of non-structural carbohydrates can alter microbial ecology and fermentation patterns [2,12,13,14]. Dietary protein, lipids, and microbiome-targeted feed additives further influence microbial metabolism and the production of biologically active metabolites [4,11,15]. As a result, dietary management has emerged as a key mechanism through which the microbiome may affect equine health and disease [9,12].

Many of the physiological effects of diet-induced microbial modulation are mediated through microbial-derived metabolites. Short-chain fatty acids produced during complex carbohydrate fermentation are important mediators of energy metabolism and intestinal homeostasis [7]. Bile acid derivatives participate in microbiota–host crosstalk and metabolic regulation [16], whereas tryptophan metabolites contribute to epithelial barrier function and immune regulation [17]. Endotoxins contribute to systemic inflammatory signaling [18], whereas nitrogenous fermentation products can affect epithelial physiology and gut health [19,20]. Collectively, these metabolites provide a mechanistic bridge connecting dietary inputs with host physiological outcomes and may therefore represent more informative biomarkers of microbial function than taxonomic composition alone.

Disruption of the nutrition–microbiota–metabolite axis has been implicated in a wide range of equine diseases. Altered microbial fermentation and metabolite production have been associated with gastrointestinal disorders such as colic, colitis, and equine gastric ulcer syndrome (EGUS) [21]. Emerging evidence further suggests potential roles for microbial metabolites in metabolic disorders including equine metabolic syndrome (EMS) and carbohydrate-associated laminitis [22]. More recently, interest has expanded beyond the gastrointestinal tract, with studies proposing potential links between intestinal microbial activity, respiratory health, and exercise-associated conditions through the gut–lung axis [23]. However, the strength of evidence varies substantially among disease categories, and many mechanistic pathways remain incompletely understood in horses.

In parallel, advances in metagenomics, metabolomics, and other multi-omics technologies are providing new opportunities to investigate microbial function at the gene, pathway, and metabolite levels [8]. These approaches have the potential to move equine nutrition research beyond descriptive microbiome characterization toward mechanistic understanding and individualized nutritional management [24].

**Figure 1:**
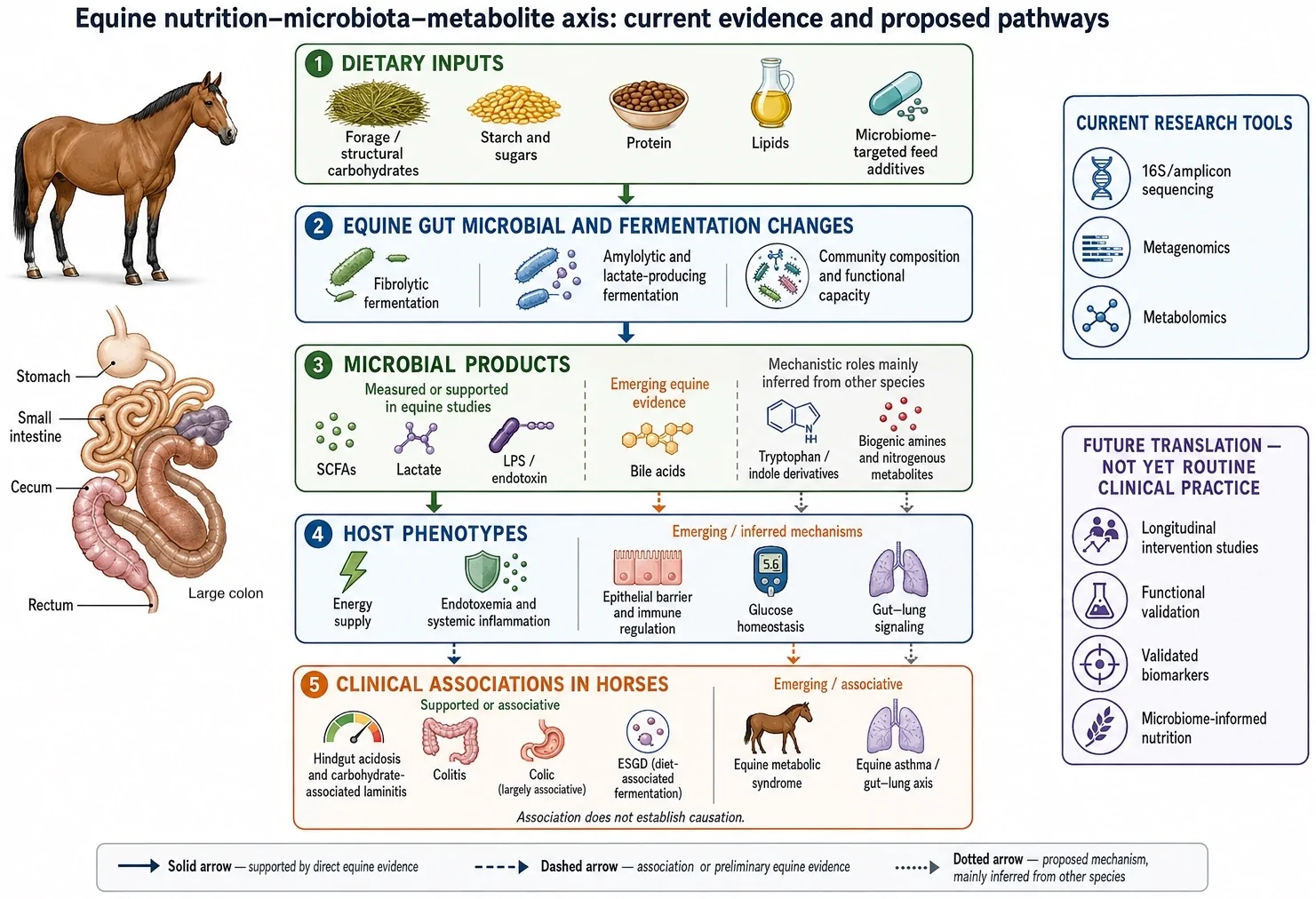
Conceptual framework illustrating the interactions among dietary factors, gut microbial function, microbial metabolites, host responses, and disease outcomes in horses. (This figure was created with the assistance of an AI-based image-generation tool and subsequently reviewed and edited by the authors).

Therefore, the objective of this review was to synthesize current evidence regarding the interactions among dietary factors, gut microbial function, microbial metabolites, and host physiology in horses. Emphasis is placed on microbial metabolites as functional mediators linking nutrition to gastrointestinal, metabolic, and respiratory health. In addition, emerging microbiome-targeted nutritional strategies and future precision nutrition approaches are discussed as potential tools for improving equine health, welfare, disease resilience, and athletic performance. The conceptual framework linking dietary factors, gut microbial function, microbial metabolites, host responses, and disease outcomes is illustrated in Figure 1.

Distinct from previous equine microbiome reviews that have focused primarily on microbial composition, dysbiosis, or dietary determinants, this review adopts an integrated nutrition–microbiota–metabolite framework. This study evaluates microbial metabolites as functional mediators linking diet with gastrointestinal, metabolic, respiratory, and exercise-associated outcomes. Equine-derived evidence is distinguished from mechanisms inferred from human, rodent, ruminant, or in vitro studies.

## 2. Methodological Strategy

This article was conducted as a narrative review supported by a structured literature search. Searches were completed in June 2026 in PubMed, Web of Science, Scopus, and Google Scholar. The final synthesis comprised 133 cited sources; the dated literature ranged from 1975 to June 2026, with one undated background source. The documents consisted predominantly of peer-reviewed original research and review articles, supplemented by consensus statements, a conference abstract, and relevant books or book chapters.

Search terms combined “horse” or “equine” with “gut microbiome,” “intestinal microbiota,” “diet,” “nutrition,” “microbial metabolites,” “short-chain fatty acids,” “bile acids,” “tryptophan metabolites,” “endotoxin,” “colic,” “colitis,” “gastric ulcer,” “lami-nitis,” “equine metabolic syndrome,” and “gut–lung axis.” Reference lists of relevant publications were screened to identify additional sources. Peer-reviewed equine studies addressing diet, microbial composition or function, metabolite production, or clinical outcomes were prioritized. Publications were excluded when they did not address the review framework or provided insufficient biological or clinical relevance. Where direct equine evidence was unavailable, mechanistic studies in other mammalian species were used to provide biological context and were explicitly distinguished from equine-specific evidence. Because of the heterogeneity in study populations, diets, sampling sites, analytical platforms, and outcomes, no quantitative meta-analysis was attempted.

### Main Findings

The findings indicate that dietary composition is a major modifiable driver of equine gut microbial function. Forage-rich diets generally support fibrolytic communities, short-chain fatty acid production, and hindgut stability, whereas excessive non-structural carbohydrate intake promotes rapid fermentation, acidification, dysbiosis, endotoxin exposure, and carbohydrate-associated laminitis. Evidence is strongest for links between the nutrition–microbiota–metabolite axis and gastrointestinal disorders, including colic and colitis, while associations with equine metabolic syndrome, respiratory disease, and the gut–lung axis remain preliminary. Probiotics, prebiotics, postbiotics, fermentable fibers, and other microbiome-targeted strategies show potential, but inconsistent formulations, small sample sizes, and methodological heterogeneity currently limit clinical translation. Standardized longitudinal studies integrating microbiome, metabolome, dietary, and clinical data are therefore required before precision nutrition can be implemented reliably in horses.

## 3. Dietary Drivers of Gut Microbial Functional Dynamics in Horses

### 3.1. Structural Carbohydrates

Structural carbohydrates, primarily cellulose, hemicellulose, pectin and lignin, constitute the evolutionary and physiological foundation of the horse’s diet. As hindgut fermenters, horses rely almost entirely on microbial carbohydrate-active enzymes (CAZymes) to degrade plant cell wall polysaccharides that escape enzymatic digestion in the foregut [1].

The equine fibrolytic community is characterized by members of the genera *Fibrobacter* and *Ruminococcus*, and the families *Lachnospiraceae* and *Ruminococcaceae*. Among these taxa, *Fibrobacter succinogenes* and *Ruminococcus flavefaciens* are recognized as the major cellulolytic microorganisms in the equine hindgut. Whereas *Ruminococcus albus* is generally detected at lower abundance in horses, ponies and donkeys. Collectively, these microorganisms initiate the degradation of cellulose and hemicellulose, generating soluble sugars and oligosaccharides that subsequently fuel downstream fermentation processes [5]. Different fibrolytic microorganisms employ distinct strategies for plant fiber degradation [5,25]. Species of *Ruminococcus* possess cellulosome-associated enzyme systems that facilitate adhesion to and degradation of plant cell walls [26,27,28,29]. In contrast, *Fibrobacter succinogenes* lacks the structural components required for classical cellulosome formation and instead utilizes fibro-slime proteins, outer membrane vesicles, and membrane-associated CAZymes to access and degrade structural carbohydrates [25]. Recent genomic analyses have revealed that horse-derived *Fibrobacter succinogenes* isolates possess extensive repertoires of glycoside hydrolases (GHs). Equine-associated strains contain a greater number of GH5 cellulases than ruminal isolates [30]. The greater GH5 enzymes representation may enhance the ability of equine strains to rapidly utilize fibrous substrates. This genomic feature may reflect adaptation to the relatively rapid passage of digesta and the hindgut-based fermentation system characteristic of equids [15].

In addition, anaerobic fungi belonging to the phylum *Neocallimastigomycota*, particularly *Piromyces* spp., contribute to fiber degradation through both enzymatic hydrolysis and physical disruption of plant tissues [31,32]. These fungi penetrate plant cell walls via rhizoidal structures, thereby increasing substrate accessibility for bacterial colonization and fermentation [31,32,33]. Although considerably less studied than bacterial populations, anaerobic fungi are increasingly recognized as important contributors to lignocellulose degradation in herbivorous mammals, including horses [32,34].

The principal products of fibrolytic fermentation are short-chain fatty acids (SCFAs), primarily acetate, propionate, and butyrate [2,6]. These metabolites provide an estimated 50–70% of the horse’s maintenance energy requirements [2,4,6]. Beyond energy provision, forage-based diets promote microbial diversity and hindgut stability [12,35,36]. Daly et al. demonstrated that horses maintained on high-forage diets exhibit greater microbial stability and higher abundances of fibrolytic taxa than horses fed concentrate-rich diets [12]. Furthermore, antibiotic administration markedly reduces cellulolytic bacterial populations while increasing opportunistic pathogens such as *Salmonella* spp. and *Clostridioides difficile*, highlighting the ecological importance of fibrolytic communities in maintaining gastrointestinal homeostasis [5]. Therefore, adequate forage intake remains the cornerstone of equine nutritional management, supporting microbial function, hindgut stability, and overall health [12,36,37].

### 3.2. Non-Structural Carbohydrates (Starch/Sugar)

Non-structural carbohydrates, including starches and simple sugars, are highly digestible energy sources widely incorporated into equine diets to support athletic performance and production demands [2]. However, when starch intake exceeds approximately 1–2 g/kg body weight per meal, the hydrolytic capacity of the small intestine may be surpassed, allowing undigested starch to enter the cecum and colon where it becomes available for rapid microbial fermentation [38].

The influx of starch into the hindgut triggers profound alterations in microbial community structure and function [12,14,35,39]. Studies have demonstrated that starch-rich diets selectively favor the proliferation of amylolytic and lactate-producing bacteria, particularly members of the *Streptococcus bovis*/*equinus* complex and *Lactobacillus* spp [21,40]. Experimental grain-overload demonstrate rapid expansion of these bacterial populations, accompanied by increases in lactate production and reductions in luminal pH [21]. As hindgut pH declines below physiological levels, fibrolytic microorganisms such as *Fibrobacter* and *Ruminococcus* are suppressed, leading to reduced fiber fermentation capacity and loss of microbial diversity [12,21]. The resulting dysbiosis is associated with increased intestinal permeability, endotoxin release, and inflammatory signaling [18,41]. Metabolomic studies have further shown that starch-rich diets alter hindgut fermentation profiles by increasing lactate and valerate concentrations while reducing beneficia SCFA production [8,42]. Consequently, excessive dietary starch represents one of the most important nutritional drivers of hindgut acidosis, microbial instability, and gastrointestinal disease in horses [2,41].

### 3.3. Protein Intake and Nitrogen Metabolism

Dietary protein is essential for growth, reproduction, tissue repair, immune function, and athletic performance in horses [43,44]. The majority of dietary protein is digested and absorbed as amino acids in the small intestine. And a proportion of undigested protein and endogenous nitrogen sources such as urea and sloughed epithelial cells enters the hindgut and becomes available for microbial fermentation [43,44]. Consequently, dietary protein intake influences not only host nitrogen metabolism but also the composition and metabolic activity of the hindgut microbiota [19].

Within the cecum and colon, proteolytic microbes including *Clostridium*, *Bacteroides*, and related anaerobic taxa degrade proteins into peptides and free amino acids [41]. These amino acids undergo subsequent deamination resulting in the production of branched-chain volatile fatty acids including, isobutyrate, isovalerate, and 2-methylbutyrate [45]. Although these metabolites can support microbial growth and nitrogen recycling, excessive proteolytic fermentation may alter hindgut homeostasis and microbial community structure [19,20].

Dietary protein levels significantly modulate the diversity and composition of the equine hindgut microbiota [46,47,48]. In Dezhou donkeys, as crude protein (CP) levels increased from 11.03% to 14.06%, observed species, Chao1, and abundance-based coverage estimator indices showed a concentration-dependent increase, suggesting that higher nitrogen availability promotes nutrient enrichment and enhances community richness [46]. Conversely, in late-gestation Mongolian mares, a medium protein level (12.04% CP) was associated with greater microbial diversity than either higher (13.25%) and lower (10.85%) protein level [47]. These findings suggest that excessive proteolytic fermentation may alter hindgut homeostasis and microbial community structure [49]. This apparent discrepancy may reflect differences in equid species, age, physiological status, basal diet composition, and the microbial diversity indices examined. Therefore, the optimal dietary protein level for maintaining hindgut microbial homeostasis likely varies according to age, physiological status, and production purpose, and remains insufficiently defined in equine populations [46,47].

Dietary protein source also appears to influence microbial composition. Studies in donkey foals have shown soybean meal supplementation, a commonly used plant-based protein source, increases the abundance of potentially beneficial genera such as *Oscillospiraceae_UCG-005*, *Akkermansia*, and *Porphyromonas* [48]. While excessive crude protein intake has been associated with the enrichment of Proteobacteria (e.g., *Acinetobacter*), which is frequently considered a microbial signature of gut dysbiosis. Conversely, low protein diets tend to favor *Bacteroidetes* and *Prevotella* spp., reflecting adaptive toward enhanced fiber utilization [47].

An additional feature of hindgut nitrogen metabolism is the ability of certain fibrolytic bacteria to utilize urea as a nitrogen source [25,50]. Genomic analyses have revealed that several equine-associated *Fibrobacter succinogenes* strains possess genes involved in nitrogen utilization, suggesting that these organisms may contribute to nitrogen conservation under conditions of limited protein supply [25,30]. This adaptation may be particularly important for horses consuming low-quality forage diets.

Beyond ammonia and branched-chain fatty acids, proteolytic fermentation generates a diverse range of nitrogenous metabolites, including biogenic amines, indoles, skatole, and other aromatic amino acid derivatives [19,20,45,51,52]. Although the physiological significance of these metabolites in horses remains incompletely understood, accumulating evidence from both equine and comparative studies suggests that excessive proteolytic fermentation may impair epithelial barrier function and contribute to low-grade intestinal inflammation [20]. The biological functions and pathological implications of these metabolites are discussed in greater detail in Section 3.

### 3.4. Dietary Lipids

Dietary lipid supplementation is widely used in equine nutrition to increase dietary energy density while reducing reliance on non-structural carbohydrates. This strategy is common in performance horses because it provides an alternative energy source without increasing the risk of starch-associated hindgut disturbances [53].

Although most dietary lipids are absorbed in the small intestine, a proportion of unabsorbed fatty acids may reach the hindgut, where they interact with the resident microbiota [54,55]. Compared with carbohydrate fermentation, microbial utilization of lipids is limited. However, hindgut microorganisms possess the capacity to modify unsaturated fatty acids through biohydrogenation pathways, converting polyunsaturated fatty acids into more saturated forms [56,57]. Most mechanistic evidence for these processes originates from ruminant systems, whereas direct evidence in horses remains comparatively limited.

Recent studies suggest that dietary lipid supplementation may subtly influence hindgut microbial composition and function [58,59,60,61]. In particular, omega-3 polyunsaturated fatty acids have attracted attention because of their anti-inflammatory properties and potential interactions with microbial metabolism [58]. However, the effects of dietary fat on the equine microbiome appear substantially less pronounced than those associated with dietary fiber, starch, or protein [59]. Consequently, current evidence suggests that the primary benefit of lipid supplementation lies in its ability to provide a concentrated energy source while reducing dependence on high-starch diets, thereby indirectly supporting hindgut stability and metabolic health [59].

## 4. Microbial Metabolites as Functional Mediators Linking Diet and Disease

Gut microbial metabolites represent the primary functional outputs of diet–microbiota interactions and serve as critical mediators linking diet with host physiology. In horses, microbial fermentation of dietary substrates within the cecum and colon generates a diverse array of metabolites that influence intestinal homeostasis, energy metabolism, immune regulation, and systemic health [9,62,63]. Compared with taxonomic descriptions of microbial communities, metabolite profiles provide a more direct reflection of microbial function and may therefore better explain the biological consequences of dietary interventions and dysbiosis [8,63,64].

### 4.1. Short-Chain Fatty Acids

In healthy forage-fed horses, acetate generally accounts for 60–70% of total SCFA production, whereas propionate and butyrate contribute approximately 20–30% and 10–20%, respectively [2,12,65]. SCFAs are rapidly absorbed across the cecal and colonic epithelium and contribute an estimated 50–70% of the horse’s maintenance energy requirements [12]. Acetate serves as an important oxidative fuel for skeletal muscle and peripheral tissues, propionate supports hepatic gluconeogenesis, and butyrate functions as the preferred energy substrate for colonocytes [6].

Beyond energy metabolism, SCFAs exert important signaling functions. Butyrate enhances epithelial barrier integrity through promotion of tight junction assembly and mucosal repair [66], while acetate and propionate participate in immune regulation through activation of G-protein-coupled receptors [67,68]. Experimental and comparative studies indicate that SCFAs suppress pro-inflammatory cytokine production, modulate regulatory T-cell responses, and contribute to maintenance of intestinal homeostasis [69,70]. Consequently, SCFAs may represent a key mechanistic link between forage-based nutrition and gastrointestinal health [2,12,66,67,68,69,70].

### 4.2. Lipopolysaccharide

Lipopolysaccharide (LPS), a structural component of Gram-negative bacterial cell walls, represents one of the most important pro-inflammatory microbial products in equine medicine [18,71]. Under physiological conditions, LPS remains confined to the intestinal lumen. However, acidosis-induced epithelial injury and microbial lysis may facilitate endotoxin translocation into systemic circulation [21,72].

Studies of equine gastrointestinal disease have consistently demonstrated associations between endotoxemia and systemic inflammatory response syndrome [18]. Elevated circulating LPS concentrations stimulate production of TNF-α, IL-1β, and IL-6, contributing to vascular dysfunction, inflammatory signaling, and tissue injury [73,74].

Although endotoxemia is most frequently associated with colic and acute gastrointestinal disease, emerging evidence suggests that chronic low-grade endotoxin exposure may also contribute to insulin dysregulation, obesity-associated inflammation, and exercise-related oxidative stress [18,75].

### 4.3. Bile Acid Metabolites

Bile acids are increasingly recognized as signaling molecules that influence host metabolism and microbial ecology [16,76]. Following hepatic synthesis and secretion into the intestine, primary bile acids may undergo extensive microbial transformation into secondary bile acids through deconjugation and dehydroxylation reactions [77,78]. These secondary bile acids interact with host receptors including farnesoid X receptor and Takeda G-protein-coupled receptor 5 [16,30], thereby influencing glucose metabolism, lipid homeostasis, inflammatory responses, and energy expenditure [79,80].

Although bile acid metabolism has been extensively investigated in humans and laboratory animals, evidence in horses remains limited [1]. Nevertheless, recent metabolomic studies suggest that bile acid pathways may contribute to microbiome-mediated regulation of metabolic health and represent a promising area for future equine research [8].

### 4.4. Tryptophan and Indole Derivatives

Dietary tryptophan serves as a substrate for both host and microbial metabolism [17,52]. Intestinal microorganisms convert tryptophan into a variety of indole derivatives, including indole, indole-3-acetic acid, indolepropionic acid, and other aryl hydrocarbon receptor (AhR) ligands [17,81]. These metabolites regulate epithelial barrier function, antioxidant defenses, and mucosal immune responses [82,83]. Activation of AhR signaling promotes intestinal homeostasis and contributes to protection against excessive inflammation [83,84].

Recent studies in other species have highlighted the importance of tryptophan-derived metabolites in gut–brain and gut–lung communication pathways [23,85,86]. Although direct evidence remains limited in horses, these metabolites may provide a mechanistic link between intestinal microbial activity, respiratory health, and exercise-associated disorders such as Exercise-induced pulmonary hemorrhage [8,23].

### 4.5. Biogenic Amines and Nitrogenous Metabolites

Proteolytic fermentation of dietary protein generates a diverse array of nitrogenous metabolites including ammonia, biogenic amines, indoles, and skatole [19]. Histidine, lysine, arginine, ornithine, and tyrosine may be converted into histamine, cadaverine, putrescine, and tyramine through microbial decarboxylation reactions [45]. At physiological concentrations, these metabolites may contribute to microbial ecology and host signaling [19,20]. However, excessive proteolytic fermentation can lead to accumulation of ammonia and biogenic amines capable of altering epithelial function and mucosal homeostasis [20,45]. Experimental evidence from humans and other animal species indicates that elevated ammonia concentrations impair epithelial barrier integrity, disrupt cellular metabolism, and promote low-grade inflammatory responses [20]. Similarly, excessive concentrations of biogenic amines may influence vascular tone, epithelial secretion, and immune activity, whereas skatole has been associated with epithelial irritation and mucosal dysfunction [45,51].

Compared with the extensive literature available in humans and laboratory animals, direct evidence regarding the biological effects of these metabolites in horses remains limited [1,41]. Nevertheless, emerging studies suggest that excessive dietary protein intake and increased proteolytic fermentation may contribute to alterations in hindgut microbial ecology and intestinal health. Consequently, biogenic amines and related nitrogenous metabolites are increasingly recognized as potential mediators linking dietary protein intake, microbial metabolism, epithelial barrier function, and gastrointestinal homeostasis in horses [19,45].

Future investigations integrating metabolomics, microbiome analyses, and functional validation studies will be required to determine the clinical significance of these metabolites and their potential roles in equine gastrointestinal and metabolic disorders [8].

## 5. Nutrition–Microbiota–Metabolite Axis in Equine Diseases

The clinical relevance of the nutrition–microbiota–metabolite axis in horses lies in its potential to explain how dietary factors influence disease susceptibility through microbial functional changes and metabolite-mediated host responses [2,4,8]. However, the strength of evidence varies substantially among diseases and metabolite classes [1,3,11]. In particular, evidence concerning metabolic disorders [75,87,88,89], respiratory diseases [90,91], and exercise-associated conditions [64,65,92,93,94] remains comparatively exploratory and is frequently derived from associative metabolomic studies, comparative animal models, or human medicine [90,91,95,96]. Therefore, the microbiota and microbial metabolites should not be interpreted as isolated causal agents in most equine diseases [11,75,88]. Rather, they should be considered functional mediators within a broader network involving diet, microbial ecology, epithelial barrier integrity, immune activation, endocrine regulation, and host metabolism [8,66,67,70]. Studying the microbiota and its metabolites may help link nutritional exposures with host phenotypes and identify potential biomarkers of dysbiosis, targets for dietary intervention, and mechanistic hypotheses requiring experimental validation [8,88,89,97].

### 5.1. Colic

Colic is one of the most important causes of morbidity and mortality in horses and is strongly influenced by feeding management, abrupt dietary changes, forage quality, concentrate intake, water availability, and intestinal motility [98]. A recent scoping review of colic risk factors identified feeding-related management practices as among the most consistently reported risk factors for equine colic [98]. In particular, sudden dietary changes, changes in hay source, increased concentrate feeding, and alterations in access to pasture were repeatedly associated with increased colic risk across multiple epidemiological studies [98]. These observations suggest that disruption of normal hindgut fermentation may represent a key mechanistic link between dietary management and gastrointestinal disease in horses.

From a nutrition–microbiota–metabolite perspective, dietary disturbances may alter hindgut fermentation by increasing the delivery of rapidly fermentable carbohydrates to the cecum and colon [2,38]. This can promote expansion of amylolytic and lactate-producing bacteria, accumulation of lactate, reduction in luminal pH, and suppression of fibrolytic microorganisms [12,21]. These changes may contribute to colic through several non-exclusive mechanisms. Excessive fermentation can increase gas production and osmotic shifts within the large intestine, potentially promoting distension, discomfort, and altered motility [41,99]. Concurrently, reduced fibrolytic fermentation and disruption of microbial cross-feeding may alter short-chain fatty acid profiles, which are important for epithelial health and hindgut homeostasis [1,9]. In more severe cases, mucosal injury and increased intestinal permeability may facilitate translocation of endotoxin and inflammatory mediators, linking local dysbiosis to systemic inflammatory responses [18].

Nevertheless, most evidence connecting microbial metabolites directly to naturally occurring colic remains associative. Current studies support the concept that dysbiosis and altered fermentation are common features of gastrointestinal disease, but further longitudinal studies are needed to determine whether metabolite changes precede colic onset, reflect disease progression, or occur secondary to intestinal dysfunction.

### 5.2. Colitis

Equine colitis is more directly linked to disruption of the intestinal microbiota and mucosal barrier than many other gastrointestinal disorders. Antimicrobial administration, abrupt dietary changes, carbohydrate overload, infectious agents, and severe systemic illness can destabilize hindgut microbial communities and reduce colonization resistance [1,100,101]. This ecological disruption may facilitate the expansion of opportunistic or pathogenic bacteria, including *Clostridioides difficile*, *Clostridium perfringens*, and *Salmonella* spp., which are among the major infectious causes of equine enterocolitis [100].

High-throughput sequencing studies provide direct evidence that horses with colitis exhibit profound fecal microbial alterations compared with healthy horses. Costa et al. reported marked differences in the fecal microbiota of horses with colitis, including increased relative abundance of Fusobacteria and reduced representation of several taxa commonly observed in healthy horses, such as members of the class *Clostridia* and the family *Lachnospiraceae* [1]. Similar findings have been reported in horses with antimicrobial-associated diarrhea, in which loss of microbial diversity was accompanied by expansion of opportunistic pathogens, particularly *Clostridioides difficile* [101]. In a study of hospitalized horses with naturally occurring colitis, significant differences in fecal microbial composition were observed between healthy and diseased horses, including reductions in fibrolytic taxa such as *Fibrobacteres* and increases in *Proteobacteria* and *Fusobacteria* [102]. Furthermore, specific microbial signatures were associated with the subsequent development of laminitis and survival status [102]. These findings support the concept that equine colitis is not simply the consequence of overgrowth of a single pathogen but is frequently associated with broader disruption of the hindgut microbial ecosystem.

Microbial-derived products and metabolites may contribute to disease progression through epithelial injury, inflammatory activation, and impaired fluid absorption. Endotoxin released from Gram-negative bacteria and toxins produced by enteric pathogens can disrupt epithelial barrier integrity, increase mucosal permeability, and stimulate innate immune responses [18]. Loss of barrier function may then facilitate systemic translocation of bacterial products, contributing to endotoxemia, systemic inflammatory response syndrome, dehydration, and, in severe cases, shock [12,103]. Dietary and metabolic disturbances may also participate in colitis pathogenesis. Excessive delivery of rapidly fermentable carbohydrates to the hindgut can promote expansion of amylolytic and lactate-producing bacteria, lactate accumulation, reduced luminal pH, suppression of fibrolytic microorganisms, and mucosal injury [21,104]. This acidotic environment may amplify epithelial barrier dysfunction and facilitate endotoxin translocation, providing a plausible mechanistic link between carbohydrate overload, microbial dysbiosis, and inflammatory injury in the large intestine [18,41].

Compared with colic, the mechanistic connection among microbiota disruption, microbial products, and clinical disease is stronger in colitis. However, it remains difficult to determine whether dysbiosis is a primary driver, a consequence of inflammation, or both. Future studies integrating microbial community analysis, metabolomics, toxin detection, epithelial barrier assessment, and host inflammatory markers are needed to clarify causal relationships [8,101,102].

### 5.3. Equine Gastric Ulcer Syndrome

EGUS includes equine squamous gastric disease (ESGD) and equine glandular gastric disease (EGGD), which are now recognized as distinct clinical entities with different anatomical locations, risk factors, and pathophysiological mechanisms [105]. Overall, EGUS is primarily associated with acid exposure, feeding management, exercise, stress, and impairment of mucosal defense mechanisms, whereas the role of the gastric microbiome remains less well established than in hindgut disorders [105,106].

From a nutrition–microbiota–metabolite perspective, the strongest evidence relates to ESGD. High-starch feeding can promote gastric fermentation by resident bacteria, including *Lactobacillus* spp. and *Streptococcus* spp., resulting in the production of volatile fatty acids (VFAs) and lactic acid [41,106]. Under acidic gastric conditions, particularly at pH values below approximately 4.0, VFAs become predominantly non-ionized and can diffuse into squamous epithelial cells. Subsequent intracellular ionization may contribute to cellular acidification, epithelial swelling, and mucosal injury, providing a plausible mechanism by which high-starch meals increase susceptibility to squamous ulceration [41,105]. This supports current nutritional recommendations to reduce large starch meals, increase feeding frequency, and maintain adequate forage intake in horses at risk of ESGD. In contrast, the role of microbial dysbiosis in EGGD remains uncertain. Studies of the equine gastric microbiome have reported differences in microbial communities between horses with and without EGGD, but these findings have not established a causal relationship [107]. Helicobacter-like organisms have been detected inconsistently in equine gastric tissue and are not currently considered a major proven cause of EGGD [106,108]. Therefore, EGUS should be discussed as a condition in which diet-associated microbial fermentation may contribute to mucosal injury, particularly in ESGD, rather than as a disease primarily driven by gastric microbial dysbiosis.

### 5.4. Equine Metabolic Syndrome

Compared with gastrointestinal diseases, evidence linking microbial metabolites to metabolic disorders in horses remains less direct, and many mechanistic hypotheses are currently extrapolated from studies in humans and other animal species. EMS is characterized by insulin dysregulation, regional or generalized adiposity, and an increased risk of endocrinopathic laminitis [22]. Although the pathogenesis of EMS is multifactorial, increasing attention has focused on the potential contribution of the gut microbiota and its metabolites to metabolic regulation [76,87,88,89].

Several studies have reported differences in fecal microbial communities between horses with EMS and metabolically healthy controls [109,110]. Elzinga et al. observed significant alterations in the fecal microbiota of horses with EMS, suggesting that microbial dysbiosis may be associated with metabolic dysfunction [75]. Similarly, more recent microbiome studies have reported differences in microbial diversity and community structure between insulin-dysregulated and metabolically healthy horses, although findings have not always been consistent across populations and study designs [109,110]. In addition, microbial-derived products such as lipopolysaccharide have been proposed as potential mediators linking intestinal dysbiosis with chronic low-grade inflammation and insulin dysregulation [109]. However, direct evidence demonstrating similar mechanisms in horses remains limited, and the contribution of endotoxin-mediated inflammation to EMS pathogenesis has not yet been fully established.

In addition, emerging metabolomic studies indicate that alterations in microbial fermentation products, bile acid metabolism, and other microbial-derived metabolites may influence glucose homeostasis and energy metabolism [87,88,89]. Nevertheless, most current evidence remains associative. Future studies integrating microbiome profiling, metabolomics, and controlled dietary interventions will be essential to determine whether microbial metabolites can serve as biomarkers, therapeutic targets, or mediators of insulin dysregulation in horses.

### 5.5. Respiratory Disease and the Gut–Lung Axis

The gut–lung axis has emerged as an important concept describing bidirectional communication between the gastrointestinal tract and respiratory system through microbial metabolites, immune signaling pathways, and systemic inflammatory mediators [90,91]. Although this concept has been extensively investigated in humans and laboratory animals, evidence in horses remains comparatively limited [23]. Nevertheless, growing interest in equine respiratory disease [23], athletic performance, and microbiome research [65,92,94] suggests that intestinal microbial ecology may influence pulmonary health through metabolite-mediated mechanisms [23,90,91].

The strongest evidence supporting a gut–lung interaction in horses currently relates to severe equine asthma. Recent studies have reported alterations in both gastrointestinal and respiratory microbial communities in affected horses, suggesting that intestinal dysbiosis may contribute to pulmonary immune dysregulation [23,111,112]. While causality has not been established, these findings are consistent with observations in humans and laboratory animals where microbial metabolites regulate respiratory immune responses. SCFAs particularly acetate, propionate, and butyrate, are considered key mediators of gut–lung communication. Studies in mice have demonstrated that SCFAs promote regulatory T-cell differentiation, suppress excessive Th2 and Th17 responses, and enhance mucosal immune tolerance [90,91].

Exercise-induced pulmonary hemorrhage (EIPH) is one of the most prevalent respiratory disorders affecting racehorses and high-performance equine athletes. Traditionally, EIPH has been attributed to extreme pulmonary vascular pressures generated during maximal exercise, resulting in stress failure of the pulmonary capillary network [113]. However, recent advances in microbiome science raise the possibility that gut-derived inflammatory signals may influence pulmonary vascular resilience and recovery [23,92,111]. During intense exercise, substantial redistribution of blood flow away from the gastrointestinal tract results in transient intestinal hypoperfusion and epithelial stress [95,96]. Similar mechanisms have been described in human endurance athletes, where exercise-induced gut dysfunction is associated with increased intestinal permeability and translocation of microbial-derived inflammatory mediators [95,96]. In horses, prolonged exercise has also been associated with alterations in fecal microbial composition and metabolite profiles, suggesting that the intestinal microbiome responds dynamically to athletic exertion [64]. However, these findings should not be directly extrapolated to EIPH, which typically occurs during high-intensity, near-maximal exercise [64].

A proposed gut–lung–performance axis suggests that diet-induced dysbiosis and impaired intestinal barrier integrity may contribute to chronic low-grade systemic inflammation [95,96]. Circulating microbial products such as endotoxin, together with altered production of anti-inflammatory metabolites including SCFAs, could theoretically influence pulmonary endothelial function, immune activation, and recovery following strenuous exercise [92,93,94,114]. Although direct evidence linking microbial metabolites to EIPH severity is currently lacking, this hypothesis provides a biologically plausible mechanism connecting nutritional management, intestinal health, and respiratory performance [113].

## 6. Nutritional Manipulation of the Equine Microbiome Function

Growing evidence suggests that dietary manipulation represents the most practical approach for modulating microbial function and metabolite production in horses. Although many microbiome-targeted interventions remain insufficiently validated, current strategies primarily focus on promoting fibrolytic fermentation, maintaining hindgut stability, and preventing dysbiosis-associated disease [2,35,115]. However, their clinical application is limited by variability among commercial products and a lack of standardized, adequately powered clinical trials in horses [116,117].

### 6.1. Probiotics, Prebiotics, and Synbiotics

Probiotics, including *Saccharomyces cerevisiae*, *Lactobacillus* spp., and *Bifidobacterium* spp., are widely used to support microbial stability during dietary transitions and periods of stress [118,119]. Prebiotics such as fructo-oligosaccharides, inulin, and mannan-oligosaccharides selectively stimulate beneficial microbial populations and may improve hindgut fermentation. Synbiotic approaches combining probiotics with complementary prebiotic substrates represent a promising strategy, although evidence for consistent clinical benefits in horses remains variable [120]. Supplementation with Saccharomyces cerevisiae has been reported to stabilize hindgut fermentation, reduce lactate accumulation, and improve fiber digestibility, particularly in horses receiving concentrate-rich diets [118,119]. In horses fed high-starch diets, live yeast supplementation increased apparent acid detergent fiber digestibility and promoted greater dry matter and neutral detergent fiber intake, suggesting improved fibrolytic activity and hindgut function [119]. Furthermore, yeast supplementation has been associated with enhanced cellulolytic enzyme activity and greater polysaccharidase activity within the hindgut microbial community [119].

### 6.2. Postbiotics

Postbiotics, defined as non-viable microbial products or metabolites that confer health benefits, have recently emerged as a promising alternative to live microbial supplementation [121,122]. Short-chain fatty acids, microbial cell components, and fermentation-derived bioactive compounds may provide direct effects on epithelial barrier function, immune regulation, and metabolic signaling without requiring successful microbial colonization [123].

Research evaluating postbiotic interventions in horses remains limited. Recent study using *Saccharomyces cerevisiae* fermentation products supplement in racehorses for 43 days, Lucassen et al. observed alterations in fecal microbial composition together with changes in circulating immune cell populations, suggesting that fermentation-derived products can influence both microbial ecology and host immune function [124]. Similarly, Ganda et al. reported that horses receiving a yeast-derived postbiotic during a transport-associated stress challenge maintained significantly more stable microbial diversity and microbiome structure than untreated controls [125]. These findings suggest that postbiotic supplementation may improve microbiome resilience under physiological stress.

### 6.3. Resistant Starch and Fermentable Fiber

Maintaining adequate forage intake remains the cornerstone of equine microbiome management. High-forage diets support fibrolytic microorganisms, promote short-chain fatty acid production, and maintain hindgut stability [9,12]. Fermentable fiber sources such as beet pulp, soybean hulls, and alfalfa products may provide energy through hindgut fermentation and reduce reliance on dietary starch [37]. Compared with fiber-rich diets, starch-rich diets are associated with marked alterations in microbial fermentation and greater risks of lactate accumulation and hindgut acidification [14,41,118]. Experimental studies have demonstrated that forage-rich diets are associated with greater microbial stability and higher abundances of fibrolytic taxa compared with concentrate-rich diets [12,40].

## 7. From Microbiome to Precision Nutrition: Emerging Concepts

Advances in microbiome research are shifting equine nutrition from descriptive analyses of microbial composition toward functional approaches that integrate microbial activity, metabolite production, and host responses. Compared with taxonomic profiling alone, multi-omics technologies—including metagenomics and metabolomics—provide greater insight into the biological mechanisms linking diet, microbial function, and disease [126].

Recent integrated microbiome–metabolome analyses have identified distinct microbial and metabolic signatures associated with intestinal injury, while large-scale metagenomic studies have revealed differences in microbial functional capacity among horses with different management systems and athletic backgrounds [8,24]. These findings support the concept that future nutritional strategies may move beyond generalized supplementation toward more individualized approaches based on microbial function and metabolite production [62,92,93]. However, most current evidence remains associative, and causal relationships between specific microbial pathways and equine diseases have yet to be established [1,11]. Future progress will require longitudinal intervention studies integrating microbiome profiling, metabolomics, dietary management, and clinical outcomes [1,8,11,93]. Ultimately, precision nutrition in horses may rely on identifying microbial and metabolic biomarkers capable of predicting disease susceptibility, dietary responsiveness, and health outcomes, thereby enabling more targeted nutritional interventions for disease prevention and performance optimization [8,92,93,127]. However, prospective validation and standardized analytical methods will be required before these biomarkers can be incorporated into routine equine practice [8,128].

## 8. Knowledge Gaps and Future Directions

Despite substantial advances in equine microbiome research, several important challenges remain [1,8]. First, most studies have focused on bacterial communities, whereas the functional contributions of fungi, archaea, bacteriophages, and other components of the equine microbiome remain poorly understood [1]. Second, many associations between microbial metabolites and disease are based on cross-sectional observations, making it difficult to distinguish causation from correlation [75]. This limitation is particularly evident in emerging areas such as the gut–lung axis, equine metabolic syndrome, and microbiome-associated respiratory disease [23]. Third, although metabolites such as short-chain fatty acids, bile acids, indole derivatives, and biogenic amines are increasingly recognized as potential mediators of host physiology, most mechanistic evidence currently originates from studies in humans and laboratory animals rather than horses [17,52,129,130]. In addition, fecal samples, although widely used as a non-invasive proxy for the distal hindgut microbiota, may not fully represent microbial communities or metabolite concentrations throughout the cecum and colon [9,131,132,133]. Differences among intestinal regions and between luminal and mucosa-associated communities should therefore be considered when interpreting fecal microbiome data.

Future research should therefore prioritize longitudinal intervention studies integrating microbiome profiling, metabolomics, dietary manipulation, and clinical outcomes [8,92,93]. Functional validation of candidate microbial pathways and metabolites will be essential to establish causal relationships and identify clinically relevant biomarkers [24]. Ultimately, combining multi-omics technologies with nutritional and clinical data may enable the development of microbiome-informed precision nutrition strategies for disease prevention, health maintenance, and performance optimization in horses [8,11].

## 9. Conclusions

The equine intestinal microbiome functions as a dynamic metabolic interface linking diet with host physiology. Increasing evidence suggests that many of the biological effects of the microbiome are mediated not only by changes in microbial composition, but also through microbial-derived metabolites that influence gastrointestinal integrity, immune regulation, metabolic homeostasis, and potentially respiratory health. Among these metabolites, short-chain fatty acids, lactate, endotoxins, bile acid derivatives, and tryptophan metabolites have emerged as key functional mediators connecting nutrition with host responses.

Current evidence provides strong support for the role of the nutrition–microbiota–metabolite axis in gastrointestinal diseases and carbohydrate-associated laminitis, whereas its involvement in metabolic disorders and the gut–lung axis remains an emerging area of investigation. As functional multi-omics technologies continue to advance, future research is expected to move beyond descriptive microbiome characterization toward mechanistic understanding and causal validation. Ultimately, integrating microbial function, metabolite profiles, and nutritional management may facilitate the development of precision nutrition strategies that improve equine health, welfare, disease resilience, and athletic performance. However, their clinical utility requires validation through standardized prospective studies.

## Data Availability

No new data were created or analyzed in this study. Data sharing is not applicable to this article.

## Abbreviations

The following abbreviations are used in this manuscript:

AhR	Aryl hydrocarbon receptor
CAZymes	Carbohydrate-active enzymes
CP	Crude protein
EGGD	Equine glandular gastric disease
EGUS	Equine gastric ulcer syndrome
EIPH	Exercise-induced pulmonary hemorrhage
EMS	Equine metabolic syndrome
ESGD	Equine squamous gastric disease
GHs	Glycoside hydrolases
GH5	Glycoside hydrolase family 5
IL-1β	Interleukin-1 beta
IL-6	Interleukin-6
LPS	Lipopolysaccharide
SCFAs	Short-chain fatty acids
Th2	T helper 2
Th17	T helper 17
TNF-α	Tumor necrosis factor alpha
VFAs	Volatile fatty acids

## References

[1] Costa M.C., Weese J.S. (2012). The Equine Intestinal Microbiome. Anim. Health Res. Rev..

[2] Julliand V., Grimm P. (2017). The Impact of Diet on the Hindgut Microbiome. J. Equine Vet. Sci..

[3] Kauter A., Epping L., Semmler T., Antao E.-M., Kannapin D., Stoeckle S.D., Gehlen H., Lübke-Becker A., Günther S., Wieler L.H. (2019). The Gut Microbiome of Horses: Current Research on Equine Enteral Microbiota and Future Perspectives. Anim. Microbiome.

[4] Chaucheyras-Durand F., Sacy A., Karges K., Apper E. (2022). Gastro-Intestinal Microbiota in Equines and Its Role in Health and Disease: The Black Box Opens. Microorganisms.

[5] Froidurot A., Julliand V. (2022). Cellulolytic Bacteria in the Large Intestine of Mammals. Gut Microbes.

[6] Bergman E.N. (1990). Energy Contributions of Volatile Fatty Acids from the Gastrointestinal Tract in Various Species. Physiol. Rev..

[7] Flint H.J., Scott K.P., Duncan S.H., Louis P., Forano E. (2012). Microbial Degradation of Complex Carbohydrates in the Gut. Gut Microbes.

[8] Whitfield-Cargile C.M., Chung H.C., Coleman M.C., Cohen N.D., Chamoun-Emanuelli A.M., Ivanov I., Goldsby J.S., Davidson L.A., Gaynanova I., Ni Y. (2024). Integrated Analysis of Gut Metabolome, Microbiome, and Exfoliome Data in an Equine Model of Intestinal Injury. Microbiome.

[9] Dougal K., Harris P.A., Edwards A., Pachebat J.A., Blackmore T.M., Worgan H.J., Newbold C.J. (2012). A Comparison of the Microbiome and the Metabolome of Different Regions of the Equine Hindgut. FEMS Microbiol. Ecol..

[10] Garber A., Hastie P., Murray J.-A. (2020). Factors Influencing Equine Gut Microbiota: Current Knowledge. J. Equine Vet. Sci..

[11] Boucher L., Leduc L., Leclère M., Costa M.C. (2024). Current Understanding of Equine Gut Dysbiosis and Microbiota Manipulation Techniques: Comparison with Current Knowledge in Other Species. Animals.

[12] Daly K., Proudman C.J., Duncan S.H., Flint H.J., Dyer J., Shirazi-Beechey S.P. (2012). Alterations in Microbiota and Fermentation Products in Equine Large Intestine in Response to Dietary Variation and Intestinal Disease. Br. J. Nutr..

[13] De Laat M.A., McGowan C.M., Sillence M.N., Pollitt C.C. (2010). Equine Laminitis: Induced by 48 h Hyperinsulinaemia in Standardbred Horses. Equine Vet. J..

[14] Raspa F., Vervuert I., Capucchio M.T., Colombino E., Bergero D., Forte C., Greppi M., Cavallarin L., Giribaldi M., Antoniazzi S. (2022). A High-Starch vs. High-Fibre Diet: Effects on the Gut Environment of the Different Intestinal Compartments of the Horse Digestive Tract. BMC Vet. Res..

[15] Li F., Kong X., Khan M.Z., Wei L., Wei J., Zhu M., Liu G., Huang B., Wang C., Zhang Z. (2025). Gut Microbiome Regulation in Equine Animals: Current Understanding and Future Perspectives. Front. Microbiol..

[16] Wahlström A., Sayin S.I., Marschall H.-U., Bäckhed F. (2016). Intestinal Crosstalk between Bile Acids and Microbiota and Its Impact on Host Metabolism. Cell Metab..

[17] Agus A., Planchais J., Sokol H. (2018). Gut Microbiota Regulation of Tryptophan Metabolism in Health and Disease. Cell Host Microbe.

[18] Moore J.N., Vandenplas M.L. (2014). Is It the Systemic Inflammatory Response Syndrome or Endotoxemia in Horses with Colic?. Vet. Clin. N. Am. Equine Pract..

[19] Windey K., De Preter V., Verbeke K. (2012). Relevance of Protein Fermentation to Gut Health. Mol. Nutr. Food Res..

[20] Blachier F., Mariotti F., Huneau J.F., Tomé D. (2007). Effects of Amino Acid-Derived Luminal Metabolites on the Colonic Epithelium and Physiopathological Consequences. Amino Acids.

[21] Milinovich G.J., Klieve A.V., Pollitt C.C., Trott D.J. (2010). Microbial Events in the Hindgut During Carbohydrate-Induced Equine Laminitis. Vet. Clin. N. Am. Equine Pract..

[22] Durham A.E., Frank N., McGowan C.M., Menzies-Gow N.J., Roelfsema E., Vervuert I., Feige K., Fey K. (2019). ECEIM Consensus Statement on Equine Metabolic Syndrome. J. Vet. Intern. Med..

[23] Leduc L., Costa M., Leclère M. (2024). The Microbiota and Equine Asthma: An Integrative View of the Gut–Lung Axis. Animals.

[24] Qin X., Xi L., Zhao L., Han J., Qu H., Xu Y., Weng W. (2025). Exploring the Distinctive Characteristics of Gut Microbiota across Different Horse Breeds and Ages Using Metataxonomics. Front. Cell. Infect. Microbiol..

[25] Neumann A.P., Suen G. (2018). The Phylogenomic Diversity of Herbivore-Associated *Fibrobacter* Spp. Is Correlated to Lignocellulose-Degrading Potential. mSphere.

[26] Ding S.-Y., Rincon M.T., Lamed R., Martin J.C., McCrae S.I., Aurilia V., Shoham Y., Bayer E.A., Flint H.J. (2001). Cellulosomal Scaffoldin-Like Proteins from *Ruminococcus flavefaciens*. J. Bacteriol..

[27] Rincon M.T., Čepeljnik T., Martin J.C., Lamed R., Barak Y., Bayer E.A., Flint H.J. (2005). Unconventional Mode of Attachment of the *Ruminococcus flavefaciens* Cellulosome to the Cell Surface. J. Bacteriol..

[28] Rincon M.T., Cepeljnik T., Martin J.C., Barak Y., Lamed R., Bayer E.A., Flint H.J. (2007). A Novel Cell Surface-Anchored Cellulose-Binding Protein Encoded by the *Sca* Gene Cluster of *Ruminococcus flavefaciens*. J. Bacteriol..

[29] Rincon M.T., Ding S.-Y., McCrae S.I., Martin J.C., Aurilia V., Lamed R., Shoham Y., Bayer E.A., Flint H.J. (2003). Novel Organization and Divergent Dockerin Specificities in the Cellulosome System of *Ruminococcus flavefaciens*. J. Bacteriol..

[30] Froidurot A., Jacotot E., Julliand S., Grimm P., Julliand V. (2024). *Fibrobacter* Sp. HC4, a Newly Isolated Strain, Demonstrates a High Cellulolytic Activity as Revealed by Enzymatic Measurements and in Vitro Assay. Appl. Environ. Microbiol..

[31] Liggenstoffer A.S., Youssef N.H., Couger M.B., Elshahed M.S. (2010). Phylogenetic Diversity and Community Structure of Anaerobic Gut Fungi (Phylum Neocallimastigomycota) in Ruminant and Non-Ruminant Herbivores. ISME J..

[32] Edwards J.E., Forster R.J., Callaghan T.M., Dollhofer V., Dagar S.S., Cheng Y., Chang J., Kittelmann S., Fliegerova K., Puniya A.K. (2017). PCR and Omics Based Techniques to Study the Diversity, Ecology and Biology of Anaerobic Fungi: Insights, Challenges and Opportunities. Front. Microbiol..

[33] Lillington S.P., Chrisler W., Haitjema C.H., Gilmore S.P., Smallwood C.R., Shutthanandan V., Evans J.E., O’Malley M.A. (2021). Cellulosome Localization Patterns Vary across Life Stages of Anaerobic Fungi. mBio.

[34] Mura E., Edwards J., Kittelmann S., Kaerger K., Voigt K., Mrázek J., Moniello G., Fliegerova K. (2019). Anaerobic Fungal Communities Differ along the Horse Digestive Tract. Fungal Biol..

[35] Hansen N.C.K., Avershina E., Mydland L.T., Næsset J.A., Austbø D., Moen B., Måge I., Rudi K. (2015). High Nutrient Availability Reduces the Diversity and Stability of the Equine Caecal Microbiota. Microb. Ecol. Health Dis..

[36] Willing B., Vörös A., Roos S., Jones C., Jansson A., Lindberg J.E. (2009). Changes in Faecal Bacteria Associated with Concentrate and Forage-only Diets Fed to Horses in Training. Equine Vet. J..

[37] Ermers C., McGilchrist N., Fenner K., Wilson B., McGreevy P. (2023). The Fibre Requirements of Horses and the Consequences and Causes of Failure to Meet Them. Animals.

[38] Fombelle A.D., Varloud M., Goachet A.-G., Jacotot E., Philippeau C., Drogoul C., Julliand V. (2003). Characterization of the Microbial and Biochemical Profile of the Different Segments of the Digestive Tract in Horses given Two Distinct Diets. Anim. Sci..

[39] Harlow B.E., Lawrence L.M., Flythe M.D. (2013). Diarrhea-Associated Pathogens, Lactobacilli and Cellulolytic Bacteria in Equine Feces: Responses to Antibiotic Challenge. Vet. Microbiol..

[40] Dougal K., De La Fuente G., Harris P.A., Girdwood S.E., Pinloche E., Newbold C.J. (2013). Identification of a Core Bacterial Community within the Large Intestine of the Horse. PLoS ONE.

[41] Al Jassim R.A.M., Andrews F.M. (2009). The Bacterial Community of the Horse Gastrointestinal Tract and Its Relation to Fermentative Acidosis, Laminitis, Colic, and Stomach Ulcers. Vet. Clin. N. Am. Equine Pract..

[42] Nilsson E., Moazzami A.A., Lindberg J.E., Jansson A. (2025). The Metabolomic Profile of a High Starch versus No Starch Diet in Athletic Horses. Sci. Rep..

[43] Mok C.H., Urschel K.L. (2020). Invited Review—Amino Acid Requirements in Horses. Asian-Australas. J. Anim. Sci..

[44] Tanner S.L., Wagner A.L., Digianantonio R.N., Harris P.A., Sylvester J.T., Urschel K.L. (2014). Dietary Crude Protein Intake Influences Rates of Whole-Body Protein Synthesis in Weanling Horses. Vet. J..

[45] Davila A.-M., Blachier F., Gotteland M., Andriamihaja M., Benetti P.-H., Sanz Y., Tomé D. (2013). Intestinal Luminal Nitrogen Metabolism: Role of the Gut Microbiota and Consequences for the Host. Pharmacol. Res..

[46] Wang Y., Diao K., Li H., Zhang C., Zhang G., Guo C. (2025). Effects of Dietary Protein Levels on Production Performance, Meat Quality Traits, and Gut Microbiome of Fatting Dezhou Donkeys. Microorganisms.

[47] Liu Y., Wang X., He Q., Wang G., Wu Z., Liu Q., Du M., Zhao Y., Bou G., Bai D. (2026). Effects of Dietary Protein on Weight Gain, Biochemical Parameters, and Gut Microbiota in Late-Gestation Grazing Mongolian Mares. Agriculture.

[48] Li Y., Zhou H., Yu J., Dong B., Li H., Zhang C., Zhang G., Guo C. (2025). Dietary Protein Sources in Concentrate Supplementation Influence Growth Performance by Manipulating Gut Microbiota and Serum Metabolites in Suckling Donkey Foals. Anim. Microbiome.

[49] Weinert J.R., Biddle A.S., Williams C.A. (2021). PSIV-17 Impacts of High Forage Crude Protein Concentrations on the Equine Fecal Microbiome. J. Anim. Sci..

[50] Maczulak A.E., Dawson K.A., Baker J.P. (1985). Nitrogen Utilization in Bacterial Isolates from the Equine Cecum. Appl. Environ. Microbiol..

[51] Smith E.A., Macfarlane G.T. (1997). Formation of Phenolic and Indolic Compounds by Anaerobic Bacteria in the Human Large Intestine. Microb. Ecol..

[52] Roager H.M., Licht T.R. (2018). Microbial Tryptophan Catabolites in Health and Disease. Nat. Commun..

[53] Jansen W.L., Van Der Kuilen J., Geelen S.N.J., Beynen A.C. (2000). The Effect of Replacing Nonstructural Carbohydrates with Soybean Oil on the Digestibility of Fibre in Trotting Horses. Equine Vet. J..

[54] Sadet-Bourgeteau S., Philippeau C., Julliand V. (2017). Effect of Concentrate Feeding Sequence on Equine Hindgut Fermentation Parameters. Animal.

[55] Bush J.A., Freeman D.E., Kline K.H., Merchen N.R., Fahey G.C. (2001). Dietary Fat Supplementation Effects on in Vitro Nutrient Disappearance and in Vivo Nutrient Intake and Total Tract Digestibility by Horses. J. Anim. Sci..

[56] Jenkins T.C., Wallace R.J., Moate P.J., Mosley E.E. (2008). BOARD-INVITED REVIEW: Recent Advances in Biohydrogenation of Unsaturated Fatty Acids within the Rumen Microbial Ecosystem. J. Anim. Sci..

[57] Kellens M.J., Goderis H.L., Tobback P.P. (1986). Biohydrogenation of Unsaturated Fatty Acids by a Mixed Culture of Rumen Microorganisms. Biotechnol. Bioeng..

[58] Nogradi N., Couetil L.L., Messick J., Stochelski M.A., Burgess J.R. (2015). Omega-3 Fatty Acid Supplementation Provides an Additional Benefit to a Low-Dust Diet in the Management of Horses with Chronic Lower Airway Inflammatory Disease. J. Vet. Intern. Med..

[59] Bronś J., Czyż K., Wyrostek A., Smoliński J., Kruszyński W., Sokoła-Wysoczańska E., Dorobisz K. (2026). The Role of Omega-3 Fatty Acids in Horses’ Nutrition—A Review. Animals.

[60] Costantini L., Molinari R., Farinon B., Merendino N. (2017). Impact of Omega-3 Fatty Acids on the Gut Microbiota. Int. J. Mol. Sci..

[61] Fu Y., Wang Y., Gao H., Li D., Jiang R., Ge L., Tong C., Xu K. (2021). Associations among Dietary Omega-3 Polyunsaturated Fatty Acids, the Gut Microbiota, and Intestinal Immunity. Mediat. Inflamm..

[62] Proudman C.J., Hunter J.O., Darby A.C., Escalona E.E., Batty C., Turner C. (2015). Characterisation of the Faecal Metabolome and Microbiome of Thoroughbred Racehorses. Equine Vet. J..

[63] Leng J., McNally S., Walton G., Swann J., Proudman C., Argo C., Emery S., La Ragione R., Eustace R. (2022). Hay versus Haylage: Forage Type Influences the Equine Urinary Metabonome and Faecal Microbiota. Equine Vet. J..

[64] Mach N., Ruet A., Clark A., Bars-Cortina D., Ramayo-Caldas Y., Crisci E., Pennarun S., Dhorne-Pollet S., Foury A., Moisan M.-P. (2020). Priming for Welfare: Gut Microbiota Is Associated with Equitation Conditions and Behavior in Horse Athletes. Sci. Rep..

[65] Li C., Li X., Liu K., Xu J., Yu J., Liu Z., Mach N., Ni W., Liu C., Zhou P. (2025). Multiomic Analysis of Different Horse Breeds Reveals That Gut Microbial Butyrate Enhances Racehorse Athletic Performance. npj Biofilms Microbiomes.

[66] Peng L., Li Z.-R., Green R.S., Holzmanr I.R., Lin J. (2009). Butyrate Enhances the Intestinal Barrier by Facilitating Tight Junction Assembly via Activation of AMP-Activated Protein Kinase in Caco-2 Cell Monolayers. J. Nutr..

[67] Koh A., De Vadder F., Kovatcheva-Datchary P., Bäckhed F. (2016). From Dietary Fiber to Host Physiology: Short-Chain Fatty Acids as Key Bacterial Metabolites. Cell.

[68] Tan J., McKenzie C., Potamitis M., Thorburn A.N., Mackay C.R., Macia L. (2014). The Role of Short-Chain Fatty Acids in Health and Disease. Advances in Immunology.

[69] Furusawa Y., Obata Y., Fukuda S., Endo T.A., Nakato G., Takahashi D., Nakanishi Y., Uetake C., Kato K., Kato T. (2013). Commensal Microbe-Derived Butyrate Induces the Differentiation of Colonic Regulatory T Cells. Nature.

[70] Smith P.M., Howitt M.R., Panikov N., Michaud M., Gallini C.A., Bohlooly-y M., Glickman J.N., Garrett W.S. (2013). The Microbial Metabolites, Short-Chain Fatty Acids, Regulate Colonic T_reg_ Cell Homeostasis. Science.

[71] MacKay R.J., Merritt A.M., Zertuche J.M., Whittington M., Skelley L.A. (1991). Tumor Necrosis Factor Activity in the Circulation of Horses given Endotoxin. Am. J. Vet. Res..

[72] Garner H.E., Coffman J.R., Hahn A.W., Hutcheson D.P., Tumbleson M.E. (1975). Equine Laminitis of Alimentary Origin: An Experimental Model. Am. J. Vet. Res..

[73] Barton M.H., Morris D.D., Norton N., Prasse K.W. (1998). Hemostatic and Fibrinolytic Indices in Neonatal Foals with Presumed Septicemia. J. Vet. Intern. Med..

[74] Leise B.S., Faleiros R.R., Watts M., Johnson P.J., Black S.J., Belknap J.K. (2011). Laminar Inflammatory Gene Expression in the Carbohydrate Overload Model of Equine Laminitis. Equine Vet. J..

[75] Elzinga S.E., Weese J.S., Adams A.A. (2016). Comparison of the Fecal Microbiota in Horses With Equine Metabolic Syndrome and Metabolically Normal Controls Fed a Similar All-Forage Diet. J. Equine Vet. Sci..

[76] Ridlon J.M., Harris S.C., Bhowmik S., Kang D.-J., Hylemon P.B. (2016). Consequences of Bile Salt Biotransformations by Intestinal Bacteria. Gut Microbes.

[77] Ridlon J.M., Wolf P.G., Gaskins H.R. (2016). Taurocholic Acid Metabolism by Gut Microbes and Colon Cancer. Gut Microbes.

[78] Joyce S.A., Gahan C.G.M. (2016). Bile Acid Modifications at the Microbe-Host Interface: Potential for Nutraceutical and Pharmaceutical Interventions in Host Health. Annu. Rev. Food Sci. Technol..

[79] Fiorucci S., Distrutti E. (2015). Bile Acid-Activated Receptors, Intestinal Microbiota, and the Treatment of Metabolic Disorders. Trends Mol. Med..

[80] Li T., Chiang J.Y.L. (2014). Bile Acid Signaling in Metabolic Disease and Drug Therapy. Pharmacol. Rev..

[81] Wikoff W.R., Anfora A.T., Liu J., Schultz P.G., Lesley S.A., Peters E.C., Siuzdak G. (2009). Metabolomics Analysis Reveals Large Effects of Gut Microflora on Mammalian Blood Metabolites. Proc. Natl. Acad. Sci. USA.

[82] Scott S.A., Fu J., Chang P.V. (2020). Microbial Tryptophan Metabolites Regulate Gut Barrier Function via the Aryl Hydrocarbon Receptor. Proc. Natl. Acad. Sci. USA.

[83] Zelante T., Iannitti R.G., Cunha C., De Luca A., Giovannini G., Pieraccini G., Zecchi R., D’Angelo C., Massi-Benedetti C., Fallarino F. (2013). Tryptophan Catabolites from Microbiota Engage Aryl Hydrocarbon Receptor and Balance Mucosal Reactivity via Interleukin-22. Immunity.

[84] Lamas B., Richard M.L., Leducq V., Pham H.-P., Michel M.-L., Da Costa G., Bridonneau C., Jegou S., Hoffmann T.W., Natividad J.M. (2016). CARD9 Impacts Colitis by Altering Gut Microbiota Metabolism of Tryptophan into Aryl Hydrocarbon Receptor Ligands. Nat. Med..

[85] Gao J., Xu K., Liu H., Liu G., Bai M., Peng C., Li T., Yin Y. (2018). Impact of the Gut Microbiota on Intestinal Immunity Mediated by Tryptophan Metabolism. Front. Cell. Infect. Microbiol..

[86] O’Mahony S.M., Clarke G., Borre Y.E., Dinan T.G., Cryan J.F. (2015). Serotonin, Tryptophan Metabolism and the Brain-Gut-Microbiome Axis. Behav. Brain Res..

[87] Von Münchow A., Torp Yttergren S., Jakobsen R.R., Luthersson N., Hansen A.K., Lindenberg F. (2023). Oligosaccharide Feed Supplementation Reduces Plasma Insulin in Geldings with Equine Metabolic Syndrome. Front. Microbiomes.

[88] Al-Ansari A.S., Duggan V., Mulcahy G., Yin X., Brennan L., Cotter P.D., Patel S.H., O’Donovan C.M., Crispie F., Walshe N. (2025). Faecal Microbiota and Serum Metabolome Association with Equine Metabolic Syndrome in Connemara Ponies. BMC Vet. Res..

[89] Donnelly C.G., Peng S., Pflieger L., Manfredi J., Coleman M., Rappaport N., Price N.D., Finno C.J. (2025). Bile Acids Segregate Metabolic Syndrome in a Cohort of 100 Deeply Phenotyped Horses. Commun. Biol..

[90] Cait A., Hughes M.R., Antignano F., Cait J., Dimitriu P.A., Maas K.R., Reynolds L.A., Hacker L., Mohr J., Finlay B.B. (2018). Microbiome-Driven Allergic Lung Inflammation Is Ameliorated by Short-Chain Fatty Acids. Mucosal Immunol..

[91] Trompette A., Gollwitzer E.S., Yadava K., Sichelstiel A.K., Sprenger N., Ngom-Bru C., Blanchard C., Junt T., Nicod L.P., Harris N.L. (2014). Gut Microbiota Metabolism of Dietary Fiber Influences Allergic Airway Disease and Hematopoiesis. Nat. Med..

[92] Plancade S., Clark A., Philippe C., Helbling J.-C., Moisan M.-P., Esquerré D., Le Moyec L., Robert C., Barrey E., Mach N. (2019). Unraveling the Effects of the Gut Microbiota Composition and Function on Horse Endurance Physiology. Sci. Rep..

[93] Mach N., Moroldo M., Rau A., Lecardonnel J., Le Moyec L., Robert C., Barrey E. (2021). Understanding the Holobiont: Crosstalk Between Gut Microbiota and Mitochondria During Long Exercise in Horse. Front. Mol. Biosci..

[94] Mach N., Midoux C., Leclercq S., Pennarun S., Le Moyec L., Rué O., Robert C., Sallé G., Barrey E. (2022). Mining the Equine Gut Metagenome: Poorly-Characterized Taxa Associated with Cardiovascular Fitness in Endurance Athletes. Commun. Biol..

[95] Costa R.J.S., Snipe R.M.J., Kitic C.M., Gibson P.R. (2017). Systematic Review: Exercise-induced Gastrointestinal Syndrome—Implications for Health and Intestinal Disease. Aliment. Pharmacol. Ther..

[96] Van Wijck K., Lenaerts K., Grootjans J., Wijnands K.A.P., Poeze M., Van Loon L.J.C., Dejong C.H.C., Buurman W.A. (2012). Physiology and Pathophysiology of Splanchnic Hypoperfusion and Intestinal Injury during Exercise: Strategies for Evaluation and Prevention. Am. J. Physiol.-Gastrointest. Liver Physiol..

[97] Li Z., Samui S., Liu J., Yang Y., Liu X., Chen Q., Li J., Gopinath D., Luo P., Shan D. (2026). Gut Microbiome and Metabolic Health: Mechanisms and Precision Interventions. Gut Microbes.

[98] Curtis L., Burford J.H., England G.C.W., Freeman S.L. (2019). Risk Factors for Acute Abdominal Pain (Colic) in the Adult Horse: A Scoping Review of Risk Factors, and a Systematic Review of the Effect of Management-Related Changes. PLoS ONE.

[99] Schoster A., Arroyo L.G., Staempfli H.R., Weese J.S. (2013). Comparison of Microbial Populations in the Small Intestine, Large Intestine and Feces of Healthy Horses Using Terminal Restriction Fragment Length Polymorphism. BMC Res. Notes.

[100] Uzal F.A., Arroyo L.G., Navarro M.A., Gomez D.E., Asín J., Henderson E. (2022). Bacterial and Viral Enterocolitis in Horses: A Review. J. Vet. Diagn. Investig..

[101] Arnold C., Pilla R., Chaffin K., Lidbury J., Steiner J., Suchodolski J. (2021). Alterations in the Fecal Microbiome and Metabolome of Horses with Antimicrobial-Associated Diarrhea Compared to Antibiotic-Treated and Non-Treated Healthy Case Controls. Animals.

[102] Ayoub C., Arroyo L.G., MacNicol J.L., Renaud D., Weese J.S., Gomez D.E. (2022). Fecal Microbiota of Horses with Colitis and Its Association with Laminitis and Survival during Hospitalization. J. Vet. Intern. Med..

[103] Destrez A., Grimm P., Cézilly F., Julliand V. (2015). Changes of the Hindgut Microbiota Due to High-Starch Diet Can Be Associated with Behavioral Stress Response in Horses. Physiol. Behav..

[104] Milinovich G.J., Trott D.J., Burrell P.C., Van Eps A.W., Thoefner M.B., Blackall L.L., Al Jassim R.A.M., Morton J.M., Pollitt C.C. (2006). Changes in Equine Hindgut Bacterial Populations during Oligofructose-induced Laminitis. Environ. Microbiol..

[105] Sykes B.W., Hewetson M., Hepburn R.J., Luthersson N., Tamzali Y. (2015). European College of Equine Internal Medicine Consensus Statement—Equine Gastric Ulcer Syndrome in Adult Horses. J. Vet. Intern. Med..

[106] Vokes J., Lovett A., Sykes B. (2023). Equine Gastric Ulcer Syndrome: An Update on Current Knowledge. Animals.

[107] Paul L.J., Ericsson A.C., Andrews F.M., Keowen M.L., Morales Yniguez F., Garza F., Banse H.E. (2021). Gastric Microbiome in Horses with and without Equine Glandular Gastric Disease. J. Vet. Intern. Med..

[108] Banse H.E., Andrews F.M. (2019). Equine Glandular Gastric Disease: Prevalence, Impact and Management Strategies. Vet. Med..

[109] Morrison P.K., Newbold C.J., Jones E., Worgan H.J., Grove-White D.H., Dugdale A.H., Barfoot C., Harris P.A., Argo C.M. (2020). The Equine Gastrointestinal Microbiome: Impacts of Weight-Loss. BMC Vet. Res..

[110] Morrison P.K., Newbold C.J., Jones E., Worgan H.J., Grove-White D.H., Dugdale A.H., Barfoot C., Harris P.A., Argo C.M. (2018). The Equine Gastrointestinal Microbiome: Impacts of Age and Obesity. Front. Microbiol..

[111] Leclere M., Costa M.C. (2020). Fecal Microbiota in Horses with Asthma. J. Vet. Intern. Med..

[112] Fillion-Bertrand G., Dickson R.P., Boivin R., Lavoie J.-P., Huffnagle G.B., Leclere M. (2019). Lung Microbiome Is Influenced by the Environment and Asthmatic Status in an Equine Model of Asthma. Am. J. Respir. Cell Mol. Biol..

[113] Hinchcliff K.W., Couëtil L.L., Knight P.K., Morley P.S., Robinson N.E., Sweeney C.R., van Erck E. (2015). Exercise Induced Pulmonary Hemorrhage in Horses: American College of Veterinary Internal Medicine Consensus Statement. J. Vet. Intern. Med..

[114] Brownlow M.A., Mizzi J.X. (2023). Pathophysiology of Exertional Heat Illness in the Thoroughbred Racehorse: Broadening Perspective to Include an Exercise-induced Gastrointestinal Syndrome in Which Endotoxaemia and Systemic Inflammation May Contribute to the Condition. Equine Vet. Educ..

[115] Collinet A., Grimm P., Julliand S., Julliand V. (2021). Sequential Modulation of the Equine Fecal Microbiota and Fibrolytic Capacity Following Two Consecutive Abrupt Dietary Changes and Bacterial Supplementation. Animals.

[116] Berreta A., Burbick C.R., Alexander T., Kogan C., Kopper J.J. (2021). Microbial Variability of Commercial Equine Probiotics. J. Equine Vet. Sci..

[117] Berreta A., Kopper J. (2022). Equine Probiotics-What Are They, Where Are We and Where Do We Need To Go?. J. Equine Vet. Sci..

[118] Medina B., Girard I.D., Jacotot E., Julliand V. (2002). Effect of a Preparation of *Saccharomyces cerevisiae* on Microbial Profiles and Fermentation Patterns in the Large Intestine of Horses Fed a High Fiber or a High Starch Diet. J. Anim. Sci..

[119] Jouany J.-P., Gobert J., Medina B., Bertin G., Julliand V. (2008). Effect of Live Yeast Culture Supplementation on Apparent Digestibility and Rate of Passage in Horses Fed a High-Fiber or High-Starch Diet. J. Anim. Sci..

[120] Perricone V., Sandrini S., Irshad N., Comi M., Lecchi C., Savoini G., Agazzi A. (2022). The Role of Yeast *Saccharomyces cerevisiae* in Supporting Gut Health in Horses: An Updated Review on Its Effects on Digestibility and Intestinal and Fecal Microbiota. Animals.

[121] Salminen S., Collado M.C., Endo A., Hill C., Lebeer S., Quigley E.M.M., Sanders M.E., Shamir R., Swann J.R., Szajewska H. (2021). The International Scientific Association of Probiotics and Prebiotics (ISAPP) Consensus Statement on the Definition and Scope of Postbiotics. Nat. Rev. Gastroenterol. Hepatol..

[122] Vinderola G., Sanders M.E., Salminen S. (2022). The Concept of Postbiotics. Foods.

[123] Aguilar-Toalá J.E., Garcia-Varela R., Garcia H.S., Mata-Haro V., González-Córdova A.F., Vallejo-Cordoba B., Hernández-Mendoza A. (2018). Postbiotics: An Evolving Term within the Functional Foods Field. Trends Food Sci. Technol..

[124] Lucassen A., Hankel J., Finkler-Schade C., Osbelt L., Strowig T., Visscher C., Schuberth H.-J. (2022). Feeding a *Saccharomyces cerevisiae* Fermentation Product (Olimond BB) Does Not Alter the Fecal Microbiota of Thoroughbred Racehorses. Animals.

[125] Ganda E., Chakrabarti A., Sardi M.I., Tench M., Kozlowicz B.K., Norton S.A., Warren L.K., Khafipour E. (2023). *Saccharomyces cerevisiae* Fermentation Product Improves Robustness of Equine Gut Microbiome upon Stress. Front. Vet. Sci..

[126] Aggarwal N., Kitano S., Puah G.R.Y., Kittelmann S., Hwang I.Y., Chang M.W. (2023). Microbiome and Human Health: Current Understanding, Engineering, and Enabling Technologies. Chem. Rev..

[127] Klein D.J., Anthony T.G., McKeever K.H. (2021). Metabolomics in Equine Sport and Exercise. J. Anim. Physiol. Anim. Nutr..

[128] Kinoshita Y., Niwa H., Uchida-Fujii E., Nukada T. (2021). Establishment and Assessment of an Amplicon Sequencing Method Targeting the 16S-ITS-23S rRNA Operon for Analysis of the Equine Gut Microbiome. Sci. Rep..

[129] Poland J.C., Flynn C.R. (2021). Bile Acids, Their Receptors, and the Gut Microbiota. Physiology.

[130] Debnath N., Kumar R., Kumar A., Mehta P.K., Yadav A.K. (2021). Gut-Microbiota Derived Bioactive Metabolites and Their Functions in Host Physiology. Biotechnol. Genet. Eng. Rev..

[131] Costa M.C., Silva G., Ramos R.V., Staempfli H.R., Arroyo L.G., Kim P., Weese J.S. (2015). Characterization and Comparison of the Bacterial Microbiota in Different Gastrointestinal Tract Compartments in Horses. Vet. J..

[132] Fliegerova K., Mura E., Mrázek J., Moniello G. (2016). A Comparison of Microbial Profiles of Different Regions of the Equine Hindgut. Livest. Sci..

[133] Arroyo L.G., Rossi L., Santos B.P., Gomez D.E., Surette M.G., Costa M.C. (2020). Luminal and Mucosal Microbiota of the Cecum and Large Colon of Healthy and Diarrheic Horses. Animals.

